# The cross-sectional average length of healthy life (HCAL): a measure that summarizes the history of cohort health and mortality

**DOI:** 10.1186/s12963-020-00220-5

**Published:** 2020-08-31

**Authors:** Markus Sauerberg, Michel Guillot, Marc Luy

**Affiliations:** 1 Vienna Institute of Demography, Austrian Academy of Sciences, Vienna, Austria; 2 Wittgenstein Centre for Demography and Global Human Capital (IIASA, OeAW, University of Vienna), Vienna, Austria; 3 Population Studies Center, University of Pennsylvania, Philadelphia, PA USA; 4 French Institute for Demographic Studies (INED), Paris, France

**Keywords:** Sullivan method, Cross-sectional average length of life, Healthy life years, Longevity, Population health

## Abstract

**Background:**

Healthy life years have superseded life expectancy (LE) as the most important indicator for population health. The most common approach to separate the total number of life years into those spent in good and poor health is the Sullivan method which incorporates the health dimension to the classic period life table, thus transforming the LE indicator into the health expectancy (HE) indicator. However, life years derived from a period life table and health prevalence derived from survey data are based on different conceptual frameworks.

**Method:**

We modify the Sullivan method by combining the health prevalence data with the conceptually better fitting cross-sectional average length of life (CAL). We refer to this alternative HE indicator as the “cross-sectional average length of healthy life” (HCAL). We compare results from this alternative indicator with the conventional Sullivan approach for nine European countries. The analyses are based on EU-SILC data in three empirical applications, including the absolute and relative level of healthy life years, changes between 2008 and 2014, and the extent of the gender gap.

**Results:**

HCAL and conventional HE differ in each of these empirical applications. In general, HCAL provides larger gains in healthy life years in recent years, but at the same time greater declines in the proportion of healthy life years. Regarding the gender gap, HCAL provides a more favourable picture for women compared to conventional HE. Nonetheless, the extent of these differences between the indicators is only of minor extent.

**Conclusions:**

Albeit the differences between HE and HCAL are small, we found some empirical examples in which the two indicators led to different conclusions. It is important to note, however, that the measurement of health and the data quality are much more important for the healthy life years indicator than the choice of the variant of the Sullivan method. Nonetheless, we suggest to use HCAL in addition to HE whenever possible because it widens the spectrum of empirical analyses and serves for verification of results based on the highly sensitive HE indicator.

## Background

Healthy life years have superseded life expectancy as the most important indicator for population health. It enables researchers to investigate, e.g. the proportion of life years spent in good/poor health, trends in life years spent in good respective poor health (the “compression-expansion-debate”), and differences between women and men [1–4]. In order to estimate the quality dimension of life years, the health expectancy indicator (HE) has been developed, which combines mortality and morbidity in a single indicator by incorporating the health dimension into the life table [5]. Even though several methods have been proposed for this purpose, the approach developed by Sullivan [6] is the most prominent one up to now [7]. It uses age-specific prevalence (proportions) of the population in the (un)healthy state, usually obtained from cross-sectional survey data, to apportion the life table person-years lived between the states of good and poor health [8].

In the application of the Sullivan method, it is frequently overlooked that life years derived from a period life table and health prevalence derived from survey data are based on different conceptual frameworks. Whereas the former reflects the life span of a hypothetical population constructed on the basis of current age-specific death rates, the latter reflects the actual health condition of real individuals [9, 10]. To overcome this conceptual mismatch between health and mortality information, it was suggested to base the Sullivan method on the “Cross-Sectional Average Length of Life” (CAL) instead of conventional period LE [11–13]. To our knowledge, this approach has not been applied empirically so far. We aim at closing this research gap by using this variant of the Sullivan approach, to which we refer as “Cross-Sectional Average Length of Healthy Life” (HCAL). Our central research question is to what extent the underlying mortality indicator, i.e. LE vs. CAL, affects the resulting estimates for healthy life years, i.e. HE vs. HCAL.

The paper is structured as follows: We start with a conceptual description of CAL in comparison to period and cohort LE to demonstrate that CAL is a combination of these two approaches. Then, we construct the HCAL indicator and discuss the difference between the mortality information in CAL and conventional LE with respect to its applicability to the Sullivan method. The empirical section starts with a description of data, followed by the presentation of our results. Here, we compare HCAL and conventional HE for nine European populations with regard to the absolute and relative level of healthy years, changes between 2008 and 2014, and differences between women and men. Finally, we discuss the advantages and disadvantages of HCAL as an alternative to HE.

## Methods

### Life expectancy and cross-sectional average length of life

Longevity measures usually follow a period or cohort concept. In a cohort life table, the observed age-specific survival probabilities define the survivorship function for a particular cohort born in time *t*. Integrating across all ages yields cohort LE at birth (${e}_0^c$), i.e. the mean age at death for this particular cohort. Formally, ${e}_0^c$ can be written as

1 $$ {e}_0^c(t)={\int}_0^{\infty }{p}_c\left(x,t\right) dx $$

with *p*_*c*_(*x*, *t*) being the probability for individuals born in time *t* to survive until age *x*. Because ${e}_0^c$ can only be calculated for extinct cohorts, and thus reflects past mortality conditions, period LE is a more convenient summary measure for current mortality levels and for tracking recent mortality trends. In this concept, the age-specific survival probabilities do not correspond to one particular birth cohort but to one particular period, i.e. constructed from the observed age-specific death rates of this calendar year. Integrating the resulting period-specific survival probabilities over all ages leads to period LE at birth (${e}_0^p$) for year *t*

2 $$ {e}_0^p(t)={\int}_0^{\infty }p\left(x,t\right) dx $$

with *p*(*x*, *t*) being the probability for individuals to survive until age *x* if they had been exposed to the survival probabilities prevailing at time *t* throughout their lives from birth to age *x*. Consequently, period LE reflects the average age at death of a hypothetical cohort under the assumption that the period-specific death rates remain unchanged over their entire life course.

The CAL concept combines the two classic concepts in the sense that it (1) refers to actual cohort mortality (i.e. it is based on longitudinal survival probabilities) and (2) corresponds to all cohorts alive in a given period (resulting in a cross-sectional summary measure of mortality experiences). CAL was originally introduced by Brouard [14] and further elaborated by Guillot [15] and Canudas-Romo and Guillot [16]. In the literature, this mixed period cohort concept has been labelled the “wedge-period perspective” [17], “cross-sectional cohort average” [18], or the “cross-sectional cohort mortality index” [19]. CAL is based on cohort survival probabilities (proportion of survivors) from birth until the last age reached at time *t*. Integrating this function across all ages yields CAL(*t*) as

3 $$ \mathrm{CAL}(t)={\int}_0^{\infty }{p}_c\left(x,t-x\right) dx $$

with *p*_*c*_(*x*, *t* − *x*) being the probability that a member of the cohort born at time *t − x* survives until age *x*. CAL(*t*) can be interpreted as a period longevity measure in the sense that it “[...] refers to a particular period *t*, but takes into account the actual mortality conditions to which cohorts present in the population at time *t* have been subject” ([15], p. 42).

**Fig. 1:**
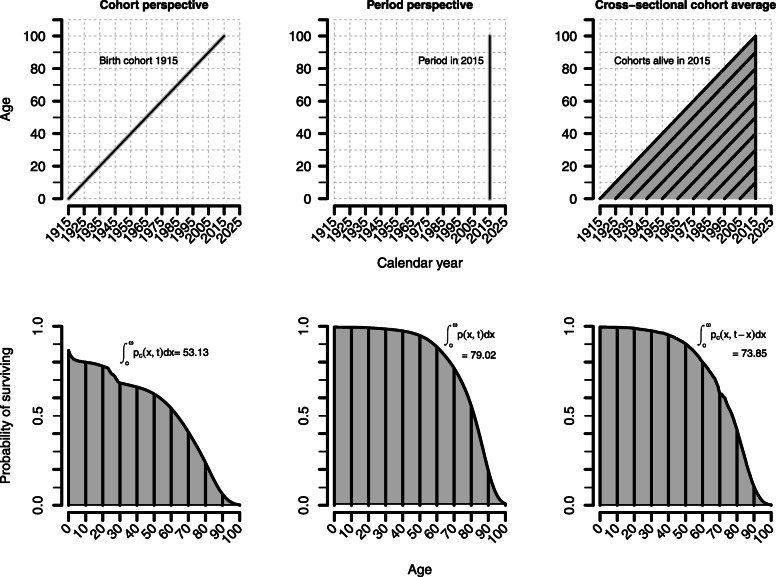
Three demographic concepts for the measurement of life years. The upper panel shows the *period perspective*, the *cohort perspective*, and the *cross-sectional cohort average concept* in a Lexis surface. The lower panel shows the empirical survivorship functions corresponding to the three concepts using data for French males in 2015. Period LE, cohort LE, and CAL are defined as the area under the curves. The three measures are given by integrating the corresponding survivorship function over all ages (depicted next to the curves)

Figure 1 illustrates the three demographic concepts using the example of French males in 2015, all constructed with data of the Human Mortality Database [20]. The upper panel shows the basic concepts in a Lexis surface. While the classic life table concept summarizes the mortality experiences of one single cohort (real or hypothetical) over its life course, CAL includes all mortality experiences experienced by cohorts alive in a given period. The lower panel shows the empirical survivorship functions corresponding to the three concepts. The areas under the curves yield cohort LE, period LE, and CAL, respectively, being 53.13 years for the cohort born in 1915, 79.02 years for the calendar year 2015, and 73.85 years for CAL in 2015. The 1915 birth cohort experienced relatively high mortality over all ages. Periods LE and CAL show similar survivorship patterns up to age 30. Then, the *p*(*x*, *t*) function of period LE is more rectangular compared to the *p*_*c*_(*x*, *t* − *x*) function of CAL. This is because the *p*_*c*_(*x*, *t* − *x*) function corresponds to actual cohorts of which many experienced higher mortality than current conditions which are reflected in period LE. The specific construction of CAL is also the reason why *p*_*c*_(*x*, *t* − *x*) is not a monotonically decreasing function. Whenever a cohort has been exposed to higher mortality conditions compared to the mortality experience of the previous (older) cohort, *p*_*c*_(*x*, *t* − *x*) will increase. This can be seen in Fig. 1 for French males born in 1945 (who reached age 70 in 2015). The proportion of cohort survivors of the 1944 birth cohort (who reached age 71 in 2015) is higher compared to the 1945 birth cohort even though they were born earlier, and therefore were longer exposed to the risk of dying.

Whether an indicator should be based on the cohort, period, or cross-sectional cohort average perspective depends on the purpose of its use. The synthetic cohort approach is a powerful tool if the aim is to examine period mortality, e.g. to investigate changes in period death rates. However, the actual survival trajectory of individuals is usually poorly captured in the hypothetical cohort scenario. For that reason, the cohort perspective is essential to analyse the real mortality experience of people in the framework of age and calendar time. However, a population at a certain time is composed of a number of cohorts. Therefore, the experience of one cohort is not representative for the entire population. This is the central advantage of the cross-sectional cohort average concept. It refers to the population as a whole by taking into account the complete mortality history of cohorts, i.e. how the actual population alive results from the cohorts’ past mortality experiences. Nonetheless, the empirical application of CAL is rare, mainly because of its high demand on the data. It has been used to study the impact of mortality on population size and growth [15], to evaluate population momentum [21], and to compare populations in terms of their mortality history [16].

### Derivation of HE and HCAL with the Sullivan method

The Sullivan method divides the total number of life years into those spent in good and in poor health. Using the life table notation, life years are expressed as person-years lived (_n_*L*_x_). The _n_*L*_x_ function allows to define *e*_o_^*p*^(*t*) as the sum of all age-specific person-years lived (divided by the period life table radix *l*_0_^p^):

4 $$ {e}_0^p(t)=\frac{1}{l_0^{\mathrm{p}}}{\sum \limits_{x=0}^{\infty}}_n{L}_x^p(t) $$

with ${}_n{L}_x^p$ being the number of person-years lived between age *x* and *x + n* in a life table for the period *t* and ${l}_0^p$ the corresponding number of people alive at age 0 (i.e. the number of newborns). CAL can be constructed from person-years lived as well. In fact, CAL is the sum of the age- and cohort-specific person-years lived divided by the cohort life table radix ${l}_0^c$, i.e. the number of newborns to which all cohorts are standardized:

5 $$ \mathrm{CAL}(t)=\frac{1}{l_0^{\mathrm{c}}}{\sum \limits_{x=0}^{\infty}}_n{L}_x^c\left(t-x-n,t-x\right) $$

with ${}_n{L}_x^c$ being the number of person-years lived between age *x* and *x + n* in the life table for the cohort born between (*t – x − n*) and (*t − x*) and ${l}_0^c$ the corresponding number of people alive at age 0.

The Sullivan method is based on the idea of applying the age-specific prevalence (proportions) of the population in an (un)healthy state to the age-specific person-years lived. In this way, the total life years in each age interval can be divided into those spent in good and in poor health. Summing up only the *healthy* person-years lived across all ages gives HE and HCAL, respectively, from:

6 $$ \mathrm{HE}(t)=\frac{1}{l_0^p}\sum \limits_{x=0}^{\infty}\left(1{-}_n{\pi}_x(t)\right)\cdot {}_n{L}_x^p(t) $$

7 $$ \mathrm{HCAL}(t)=\frac{1}{l_0^c}\sum \limits_{x=0}^{\infty}\left(1{-}_n{\pi}_x(t)\right)\cdot {}_n{L}_x^c\left(t-x-n,t-x\right) $$

with _n_π_x_ being the age-specific prevalence (proportion) of poor health in the age interval *x* to *x + n* at time *t*. The proportion of healthy life years on total life years is given by the ratios HE/LE and HCAL/CAL, respectively. Alternatively, HE and HCAL can be derived directly from the corresponding survivorship functions by weighting the survival probabilities with the population proportions of individuals being in good health. Integrating the derived functions across all ages yields HE and HCAL in continuous time from

8 $$ \mathrm{HE}(t)={\int}_0^{\infty }p\left(x,t\right)\cdotp \left(1-\pi \left(x,t\right)\right) dx $$

9 $$ \mathrm{HCAL}(t)={\int}_0^{\infty }{p}_c\left(x,t-x\right)\cdotp \left(1-\pi \left(x,t\right)\right) dx $$

**Fig. 2:**
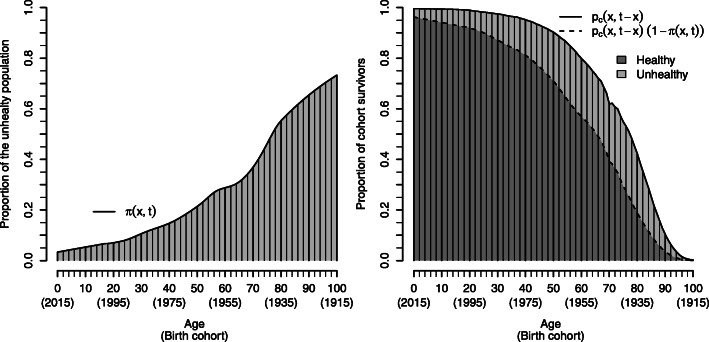
Combining cohort survivorship with proportions of individuals being in the healthy state. The vertical lines on the left side give the share of unhealthy individuals in each cohort in 2015 on the basis of data for French males, i.e. the *π*(*x*, *t*) function. The right side shows the corresponding proportions of cohort survivors *p*_*c*_(*x*, *t* − *x*). Combining these two quantities gives the proportion of being both, healthy and alive in 2015, defined as *p*_*c*_(*x*, *t* − *x*) · (1 − *π*(*x*, *t*)). Accordingly, only the dark grey shaded vertical lines refer to cohort survivors being in good health. The remaining light-shaded lines give the unhealthy share of cohort survivors

Equations 8 and 9 demonstrate that HE and HCAL solely differ in terms of the underlying survivorship function (*p*(*x*, *t*) vs. *p*_*c*_(*x*, *t* − *x*)), while the *π*(*x*, *t*) function remains the same for both measures. The combination of *p*_*c*_(*x*, *t* − *x*) with *π*(*x*, *t*), i.e. HCAL, is illustrated in Fig. 2 with data for French males in 2015. Each of the vertical lines in the right panel corresponds to a proportion of cohort survivors. The left shows the proportion of poor health according to the EU-SILC data [22]. For example, about 80% of the 1955 birth cohort survived up to 2015 (i.e. reached age 60) and approximately 30% of the same birth cohort reported to be mildly or strongly limited in 2015. Combining these two quantities gives the probability of being both healthy and alive in 2015: 0.8 · (1 – 0.3) = 0.56. The age-specific survival in good health (i.e. free of limitations) is shaded in dark grey.

Since both the *p*_*c*_(*x*, *t* − *x*) and the *π*(*x*, *t*) function correspond to the same group of individuals, HCAL creates a consistent combination of mortality and health quantities. By contrast, combining the *p*(*x*, *t*) function with the *π*(*x*, *t*) function, the conventional Sullivan method procedure (Eq. 8) combines survival probabilities corresponding to *one hypothetical* cohort with the health state-specific prevalence of *several real cohorts*. Strictly speaking, this results in a probability, which is neither reflecting healthy survival in a synthetic cohort fashion nor in a real cohort perspective. A detailed description of the particular implications for HE and HCAL resulting from the different conceptual approaches can be found in the Appendix.

### Data sources for single age-specific mortality and prevalence

We estimate HE and HCAL with data for Denmark, Finland, France, Germany, Italy, the Netherlands, Norway, Sweden, and the UK for the years 2008 to 2014. To test whether the indicators provide different results, we compare the corresponding country-specific estimates with regard to the total level of healthy life years, genders differences, and changes over time. The HE and HCAL estimation requires single age-, period-, and cohort-specific death rates and single age-specific proportions of the (un)healthy population observed in the given country and year.

Age-specific death rates for estimating LE and CAL were taken from the Human Mortality Database (HMD). For the UK, HMD data is available from 1922 onwards. However, calculating (H)CAL in 2005 requires data beginning in 1905 (defining age 100 as the highest age). To obtain mortality rates before the year 1922, we combined HMD data for the UK with HMD data for England and Wales which is available from 1841 onwards. For Germany, we used cohort mortality data from Destatis [23] because HMD provides German mortality data only from 1956 onwards. While Destatis publishes cohort life tables, the HMD provides cohort death rates only for cohorts that have lived at least 30 years (from age 0 to 29). Yet, period life tables are available also for more recent years. Therefore, we reconstructed the cohort survivorship for the (H)CAL calculation for all countries but Germany by combining the age-specific death rates of the period life tables longitudinally along the cohorts’ life course (see supplementary material). This technique has already been used in previous empirical estimations of CAL [16].

Age-specific prevalence data was taken from the European Union Statistics on Income and Living Conditions (EU-SILC) [22]. We defined “being healthy” on the basis of the “Global Activity Limitation Indicator” (GALI). GALI has been developed for providing a harmonized health indicator for monitoring population health in Europe [24] and refers to the question: “For at least the past six months, to what extent have you been limited because of a health problem in activities people usually do?” with the three answer categories “strongly limited”, “limited, but not strong”, and “not limited”. We defined being in the healthy state if respondents reported to be “not limited”. Unfortunately, the harmonization of GALI is still imperfect, hampering the comparison of HE estimates between countries and over time [25]. Previous research found that health indicators are sensitive with respect to the mode of data collection [26], the choice of the survey [27], and the wording of the health survey question [28]. Our selection of countries and time span was therefore driven by avoiding any substantial breaks in the time series and by choosing countries with the required mortality data available in the HMD (besides Germany for which data was taken from Destatis).

The country-specific sample sizes and the prevalence of being unhealthy using GALI are presented in Table 1 (separated by gender). Some data problems become apparent in these figures. For example, the prevalence of being unhealthy decreased strongly in Sweden between 2013 and 2014, and we, therefore, excluded Sweden from the time trend analysis. In Norway and Finland, we find extreme outliers in the prevalence values in 2011 and 2013, respectively, while for Italy, no EU-SILC data is available in 2010. These breaks and outliers are also mentioned in the Eurostat database [29], indicating that we cannot analyse the full time span 2008 to 2014, but at least we can compare the years 2008 and 2014. Because the health data is highly fluctuating between single age groups, we applied the R package “MortalitySmooth”. The package has been developed for smoothing count data, which can be assumed to be Poisson-distributed [30] and provides two smoothing functions: “Mort1Dsmooth” assumes smoothness in a one-dimensional (over age) way and “Mort2Dsmooth” for a two-dimensional setting (over ages and years). We applied “Mort2Dsmooth” to the data for countries without a break or an outlier between 2008 and 2014 (France, the UK, Denmark, the Netherlands, and Germany). The remaining countries (Finland, Norway, Italy, and Sweden) were smoothed in a one-dimensional way. Single age-specific proportions of being unhealthy were derived from the smoothed health data. In order to take into account also the uncertainty from the survey sample size, we approximated single age-specific standard errors by using the approximation formula ([8], p. 27), i.e. applying the smoothed proportions of being unhealthy to the observed number of persons in the corresponding age intervals. These standard errors were used to approximate 95% confidence intervals for HE and HCAL estimates which are presented in Tables 4 and 5. As to be expected, the uncertainty in single age-specific prevalence data is substantial, and we do not find statistically significant differences between HE and HCAL in an any of our empirical analyses. Therefore, we compare the two indicators for healthy life years without confidence intervals in the following section. We come back to this issue at the end of the paper when we discuss the properties of HE and HCAL.

**Table 1:** Total sample size *N* (unweighted) and total prevalence of being unhealthy 휋 (weighted) for nine European countries from 2008 to 2014

			EU-SILC survey year						
			2008	2009	2010	2011	2012	2013	2014
**Denmark**	Females	***N***	3019	3101	3072	2655	2737	2784	2959
	Males		2758	2765	2794	2477	2552	2635	2798
	Females	**휋**	30.71	30.66	29.59	31.58	30.68	31.70	31.37
	Males		23.65	24.42	23.46	21.93	27.24	26.72	28.57
**Finland**	Females	***N***	5175	5050	5423	4512	4854	5383	5418
	Males		5128	4912	5267	4586	4885	5371	5405
	Females	**휋**	34.99	36.17	37.11	37.56	40.82	47.84	39.28
	Males		29.18	29.51	29.18	30.82	32.67	40.61	30.97
**France**	Females	***N***	10,473	10,568	10,944	11,132	11,771	10,803	11,113
	Males		9535	9545	9944	10164	10742	9782	9985
	Females	**휋**	25.08	26.61	27.23	27.10	26.86	26.99	27.01
	Males		21.07	21.36	23.05	22.47	22.84	22.67	22.41
**Italy**	Females	***N***	22,635	22,072	NA	20,392	20,325	19,039	20,409
	Males		20,741	20,087	NA	18,564	18,475	17,324	18,435
	Females	**휋**	31.19	30.35	NA	31.59	32.49	32.95	31.75
	Males		23.62	22.97	NA	24.07	26.21	26.69	25.93
**Germany**	Females	***N***	12,579	12,323	12,191	12,497	12,181	11,671	11,715
	Males		11,547	11,363	11,211	11,548	11,272	10,709	10,780
	Females	**휋**	36.44	35.56	35.50	36.14	38.03	38.80	40.17
	Males		33.68	32.82	32.42	33.52	34.87	34.83	36.78
**Netherlands**	Females	***N***	5667	5274	5494	5679	5479	5384	5464
	Males		4648	4443	4628	4794	4667	4706	4680
	Females	**휋**	34.14	33.94	33.50	34.79	35.91	39.69	36.74
	Males		24.68	25.81	26.35	23.60	24.73	27.44	24.95
**Norway**	Females	***N***	2632	2587	2457	2054	2820	2838	3490
	Males		2853	2762	2704	2343	3158	3107	3782
	Females	**휋**	21.79	21.39	20.03	26.45	17.52	22.07	21.69
	Males		13.40	14.87	14.87	18.54	11.99	14.16	12.49
**Sweden**	Females	***N***	3834	3891	3713	3512	3482	3165	2933
	Males		3612	3649	3451	3193	3136	3025	2834
	Females	**휋**	29.29	26.91	26.66	27.88	27.28	27.22	16.95
	Males		21.44	19.45	20.01	21.27	20.82	20.10	10.55
**UK**	Females	***N***	8725	8081	7827	7728	9688	9716	9466
	Males		7816	7278	6970	6949	8648	8692	8437
	Females	**휋**	20.74	21.68	22.23	23.55	23.63	23.16	24.52
	Males		18.22	18.91	19.18	19.42	19.70	19.85	21.43

The table provides the total sample size (*N*) in the EU-SILC for each country and year (separated for males and females). The corresponding proportions of the unhealthy population (π) are based on the weighted survey sample. Data is not available (NA) for Italy in 2010. Source: EU-SILC data (own calculations)

## Results

### Level of healthy life years estimated with HCAL and conventional HE

Table 2 shows the estimates LE, HE, CAL, and HCAL for the nine European countries, separately for females and males. As expected, CAL is lower than LE in each country and for each gender group. This results from the fact that CAL includes also (higher) historical death rates, whereas LE is solely build up from recently observed (relatively lower) death rates. Interestingly, the differences between HE and HCAL is smaller than the differences between LE and CAL. This relationship reverses in relative terms, however. The ratio HCAL/CAL is slightly higher than the ratio HE/LE in all nine countries (and for both genders). This is due to the relative difference between the *p*(*x*, *t*) function and the *p*_*c*_(*x*, *t* − *x*) function. In relative terms, the *p*_*c*_(*x*, *t* − *x*) function is higher at young ages and lower at older ages compared to the *p*(*x*, *t*) function. In other words, the relative number of deaths is higher at young ages and lower at older ages on the basis of CAL. Since the prevalence of individuals in the unhealthy state is usually low in younger ages but increases with age, HCAL provides higher proportions of healthy life years than conventional HE (see Appendix for more details). Especially, Italian, French, and German males show a (comparatively) large gap between the ratios HE/LE and HCAL/CAL because these populations experienced high mortality in the past, particularly during the World War II. For example, Italian males spend 81.06% of their total life years in good health on the basis of HCAL, whereas the proportion of healthy life years is only 78.44%. Including the mortality history of cohorts results also in a different country ranking on the basis of CAL. While LE ranks Italy (for both genders) relatively high, CAL favours Sweden and Norway. However, HE and HCAL rank the nine analysed countries similar for females and males, the ranking changes only slightly. This is because the prevalence of activity limitations varies substantially between countries, compensating most mortality differences between LE and CAL.

**Table 2:** LE, HE, CAL, and HCAL in absolute and relative terms for nine European countries in 2014

	Denmark	Finland	France	Germany	Italy	Netherlands	Norway	Sweden	UK
**LE**									
Females	82.65	83.83	85.37	83.31	85.11	83.28	84.07	84.03	82.96
Males	78.56	78.12	79.27	78.43	80.54	79.87	80.02	80.35	79.24
**HE**									
Females	58.51	56.56	65.29	54.43	63.28	57.31	69.19	72.52	64.36
Males	59.17	58.91	63.50	55.00	63.18	62.41	71.18	73.54	64.19
**% HE/LE**									
Females	70.79	67.46	76.48	65.33	74.35	68.82	82.30	86.30	77.58
Males	75.31	75.41	80.11	70.12	78.44	78.14	88.95	91.52	81.00
**CAL**									
Females	78.52	79.83	80.75	79.07	79.07	80.25	80.93	81.20	79.28
Males	73.93	72.84	73.54	73.26	73.76	75.55	75.74	76.62	74.90
**HCAL**									
Females	56.01	54.78	62.93	52.88	60.79	55.83	66.95	70.44	62.38
Males	56.23	56.24	60.22	52.67	59.79	59.95	67.88	70.55	61.63
**% HCAL/CAL**									
Females	71.32	68.62	77.93	66.88	76.89	69.57	82.73	86.74	78.68
Males	76.06	77.21	81.89	71.89	81.06	79.35	89.62	92.08	82.29

The table provides estimates of LE, HE, CAL, and HCAL in absolute and relative terms for Denmark, Finland, France, Germany, Italy, the Netherlands, Norway, Sweden, and the UK in 2014 (separated for males and females). Source: HMD and EU-SILC data (own calculations)

### Changes in healthy life years over time: compression vs. expansion of morbidity

**Fig. 3:**
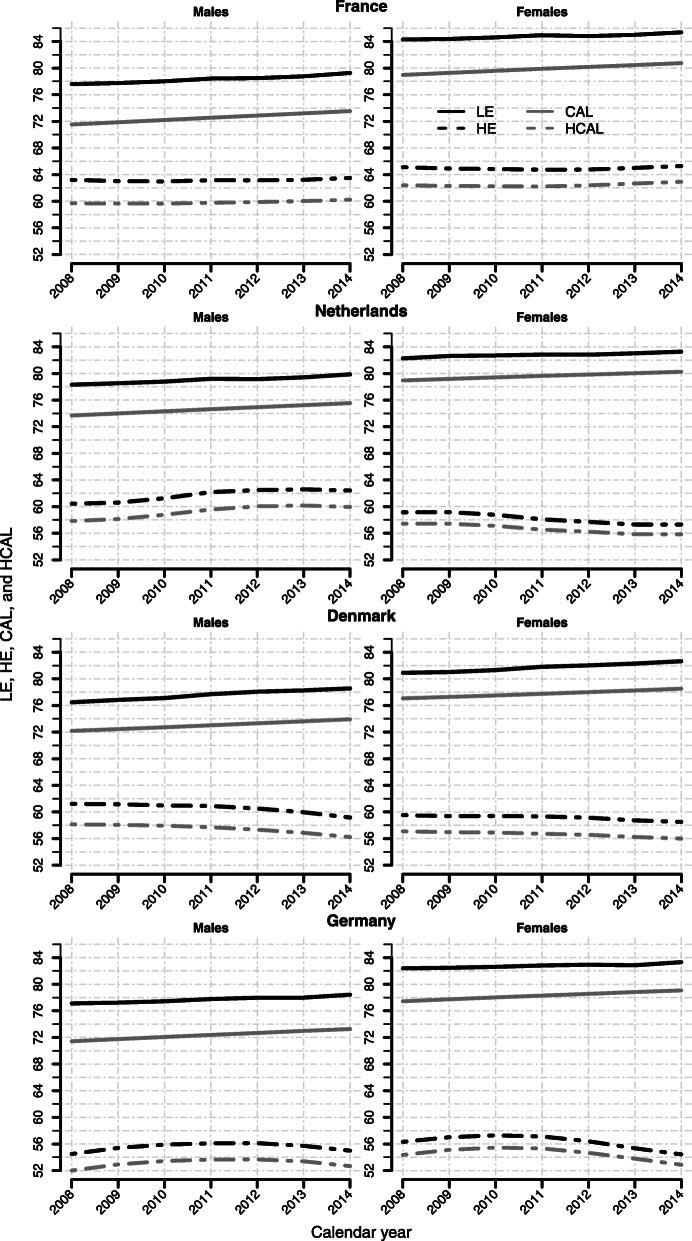
Time trend in LE, HE, CAL, and HCAL from 2008 to 2014. The figure shows how LE (black solid line), HE (black dashed line), CAL (grey solid line), and HCAL (grey dashed line) changed between 2008 and 2014. The time trends are presented for France, the Netherlands, Denmark, and Germany (males on the left side of the figure and females on the right side)

Figure 3 shows changes in LE, HE, CAL, and HCAL from 2008 to 2014 for France, the Netherlands, Denmark, and Germany, separated by gender. While CAL increases in a more or less linear fashion, LE shows some fluctuations over time. The robust trend in CAL results from the fact that it is based on a large number of age-specific death rates, and thus, it is not much affected by short-term fluctuations in period mortality (for more details see [15]). Nonetheless, the trend in HCAL is not as linearly increasing as the trend in CAL. Instead, it follows the trend in HE, indicating that prevalence data is the driving force in the corresponding time trend in healthy life years and that the choice of the basic survival function does not matter significantly.

Moreover, CAL is increasing faster than LE over time. While LE for French males increases between 2008 and 2014 by 1.67 years, CAL rises by about 2 years in the same period (see Table 3). Consequently, the increase in HCAL is also higher than the increase in HE (0.52 years vs. 0.29 years).

**Table 3:** Difference in the estimates of LE, HE, CAL, and HCAL in absolute and relative terms for eight European countries from 2008 to 2014

	Denmark	Finland	France	Germany	Italy	Netherlands	Norway	UK
**Change in LE**								
Females	1.74	0.84	1.09	0.93	0.99	1.01	1.13	1.30
Males	2.08	1.81	1.67	1.32	1.57	1.55	1.71	1.70
**Change in HE**								
Females	− 1.01	− 3.80	0.19	− 1.88	0.84	− 1.83	− 0.07	− 0.75
Males	− 2.06	0.54	0.29	0.50	− 0.02	1.98	1.27	0.77
**Change in HE/LE**								
Females	− 2.77	− 5.27	− 0.76	− 3.03	0.12	− 3.07	− 1.21	− 2.15
Males	− 4.75	− 1.08	− 1.36	− 0.55	− 1.59	0.98	− 0.32	− 0.77
**Change in CAL**								
Females	1.45	1.84	1.77	1.63	2.09	1.30	1.22	1.68
Males	1.75	2.14	2.02	1.84	2.36	1.86	1.72	1.99
**Change in HCAL**								
Females	− 1.07	− 3.25	0.54	− 1.47	1.40	− 1.60	0.07	− 0.26
Males	− 1.90	0.63	0.52	0.67	0.60	2.12	1.45	1.19
**Change in HCAL/CAL**								
Females	− 2.73	− 5.79	− 1.07	− 3.30	− 0.27	− 3.18	− 1.18	− 2.03
Males	− 4.48	− 1.44	− 1.58	− 0.91	− 1.85	0.87	− 0.12	− 0.61

The table gives the difference between the estimates in 2008 and 2014 for LE, HE, CAL, and HCAL in absolute and relative terms in Denmark, Finland, France, Germany, Italy, the Netherlands, Norway, and the UK (separated for women and men). Source: HMD and EU-SILC data (own calculations)

Note that differences in the increase in the total number of life years according to CAL and LE affect also the trend in the proportion of healthy life years, i.e. the ratio HCAL/CAL and the ratio HE/LE. In general, the increase in CAL exceeds the increase in LE between 2008 and 2014, resulting in higher decreases in the HCAL/CAL ratio. Denmark appears as a special case because both males and females show higher gains in LE compared to CAL. Accordingly, the reduction in the proportion of healthy life years is more pronounced on the basis of conventional HE.

Nonetheless, the proportions HCAL/CAL and HE/LE largely agree on the direction of the trend in healthy life years, i.e. whether we observe an expansion or compression of morbidity. The only exception is Italian females. Whereas the HE/LE ratio indicates a relative increase in healthy life years, i.e. relative compression of morbidity, the HCAL/CAL ratio suggests a slight trend in the direction of relative morbidity expansion.

### Gender differences in healthy life years

**Fig. 4:**
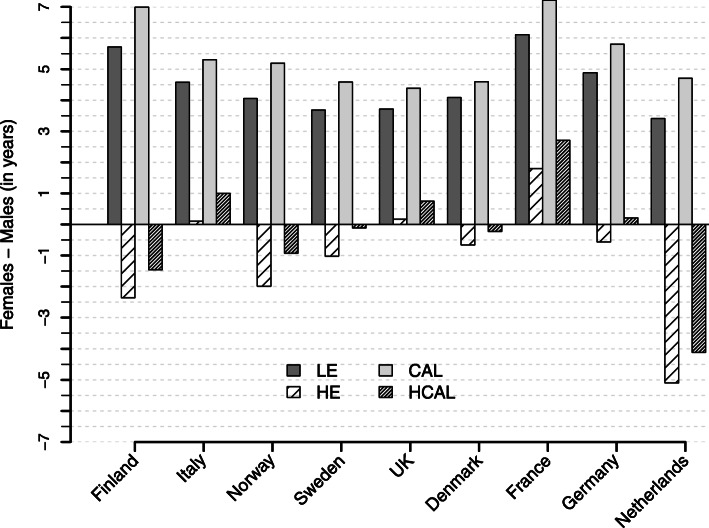
Gender gap in LE, HE, CAL, and HCAL in 2014. The figure shows the difference between males and females in LE (dark grey bars), HE (less-hatched bars), CAL (light grey bars), and HCAL (more-hatched bars) for Finland, Italy, Norway, Sweden, the UK, Denmark, France, Germany, and the Netherlands in 2014

Both mortality indicators (LE and CAL) show a female advantage in the total number of life years for all analysed populations (see Fig. 4). This gender gap is larger for CAL, indicating that the difference between male and female mortality was higher in the past compared to recent years. As a consequence, the gender gap in healthy life years according to HCAL is weighted stronger in the direction of females, i.e. HCAL provides either a larger female advantage or a smaller female disadvantage compared to HE. The differences between HCAL and HE in the extent of the gender gap differ, therefore, depending on whether females or males have a higher number of healthy life years. In Finland, Norway, Sweden, Denmark, and the Netherlands, males show a lower age-specific prevalence of activity limitation compared to women. Consequently, the gender gap is larger based on conventional HE in these populations. Yet, in countries where the age-specific prevalence of activity limitation is lower among females (Italy, the UK, and France), the gender gap is larger according to HCAL. In Germany, we find a specific situation in which HE and HCAL appear as contradictive in terms of the direction of the gender gap. The female survival advantage in CAL is large enough to compensate their higher age-specific prevalence of activity limitations, resulting in more healthy life years for females in HCAL, whereas conventional HE gives more healthy life years for males.

**Fig. 6:**
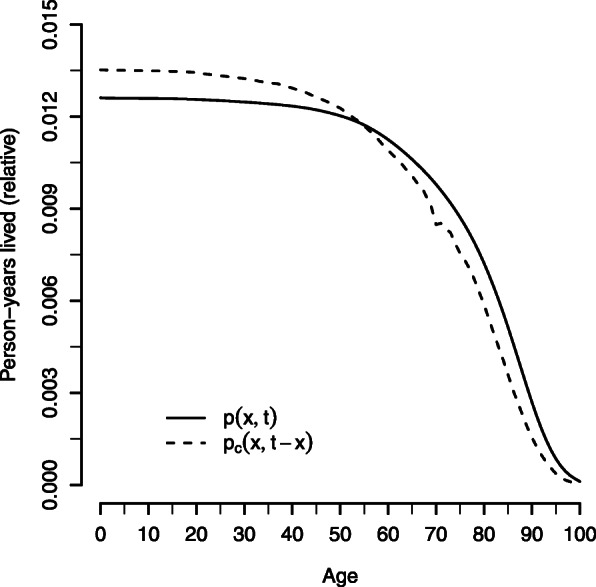
Person-years lived in relative terms on the basis of period LE and CAL. The figure shows the relative number of age-specific person-years lived between age zero and 100, i.e. the share of age-specific person-years lived on the total number of person-years lived, using the concept of period LE (solid line) and CAL (dashed line)

In relative terms, the proportion of healthy life years on total life years is higher for males in all nine countries. This male advantage is larger according to HCAL than according to HE (see Table 2). As mentioned above, differences between HE and HCAL in relative terms result from the relative difference between the *p*(*x*, *t*) function and the *p*_*c*_(*x*, *t* − *x*) function, leading to an (un)favourable age-specific weighting scheme. The comparatively high mortality for males measured with CAL promotes a situation in which higher weights are assigned to the young ages with low prevalence of being unhealthy (see Fig. 6).

## Discussion

We started this paper with a description of three different concepts for measuring longevity in a population. We have concluded that only cohort LE is an appropriate choice if the aim is to examine the real-life course experience of people in the framework of age and calendar time. However, the concepts of period LE and CAL are more convenient for monitoring health and mortality on the population level as they summarize the mortality information of an entire population, instead of focusing exclusively on a specific group of individuals. In addition, CAL and period LE are more timely than cohort LE because they can be estimated also for recent periods. However, CAL includes a high proportion of historic death rates, while period LE solely reflects recent mortality rates (see [18] for more details).

Whereas period LE reflects the life course of one hypothetical cohort by linking together a set of age-specific death rates observed in a given period, CAL summarizes the complete mortality experience of all actual living cohorts from theirs birth until the current period. A specific feature of CAL is that it takes into account the natural process of survival, i.e., the overall mortality in a certain year or period is conceptualized as the product of past exposures and health behaviour that have accumulated over the entire life span of the currently living cohorts [31]. Therefore, an observed increase in CAL between two points in time corresponds to the factual longevity gains experienced by individuals present in the given population. By contrast, trends in period LE are more difficult to interpret and might be distorted by several effects such as cohort and tempo effects or heterogeneity [19, 32]. For example, the stagnation and rise seen in Danish women’s period LE has been attributed to specific cohorts rather than to changes in period mortality conditions [33], and public health researchers are currently investigating to which extent the recent observed stalling in period LE in the UK and Europe represents a “real” deterioration of population health [34].

Period LE and CAL can be extended to HE and HCAL by applying the Sullivan method. While HCAL links the proportions of healthy individuals observed in a given population to the corresponding proportions of cohort survivors, conventional HE combines the health information of real cohorts with the survival trajectory of a hypothetical cohort. These features make CAL a more appropriate basis for the estimation of healthy life years with the Sullivan method. However, CAL is less affected by changes in recent death rates and increases also in years where period LE decreases. Therefore, one could argue that conventional HE estimates are more timely than HCAL estimates.

It is important to note that the Sullivan method itself has been criticized for producing misleading results regarding monitoring changes in population health [35–37]. The main argument of these critiques focused on using prevalence instead of incidence data. In terms of health, prevalence reflects the proportion of individuals in the unhealthy state at a given point in time. This includes individuals who transitioned from healthy to unhealthy in the observation period as well as those who experienced this transition already in the past. The incidence of being unhealthy, however, refers exclusively to individuals who experienced transitions during the given calendar year (or period). As a consequence, health indicators estimated with the Sullivan method cannot capture a sudden short-term change in population health regardless of the choice of the mortality information [38]. Nevertheless, prevalence-based indicators such as HE and HCAL are convenient for measuring the current health composition of a population, i.e. the actual proportion of healthy/unhealthy individuals in a population [39].

The use of health prevalence data makes HCAL the conceptually more coherent indicator for monitoring population health. The data demands are, however, somewhat higher for HCAL than for HE. This leads to the question, whether results between HCAL and HE differ to an extent that justifies this higher data demand. Therefore, we compared the HCAL indicator to conventional HE in three empirical applications for nine European countries. In general, HCAL is lower in absolute terms and slightly larger in relative terms due to incorporating (higher) historical death rates. Our examination of the gender gap in health and mortality shows that HCAL provides a larger female advantage in healthy life years than conventional HE. This finding illustrates the implication of using period mortality (in LE) instead of cohort mortality information (in CAL). The gender-specific differences in the prevalence of being unhealthy can be (partly) attributed to the different health risks and exposures, i.e. the mortality, experienced by women and women over their life span [40, 41]. In the case of HCAL, prevalence is related to the corresponding mortality history, which had considerable higher levels for men compared to women. Conventional HE, on the other hand, relates the prevalence to current period death rates, which show lower gender differences in mortality than actual cohorts have been experienced over their life courses.

The probably most discussed question in health research is, whether gains in longevity are spent primarily in good or poor health, in the context of the so-called “expansion vs. compression of morbidity debate”. The ratio of LE/HE respective CAL/HCAL is particularly relevant in this context because it shows the relative share of healthy life years on total life years. In general, we found larger gains in CAL compared to period LE between 2008 and 2014, resulting in a slightly faster decreasing HCAL/CAL ratio compared to the LE/HE ratio. This can be interpreted as a stronger relative expansion of morbidity on the basis of HCAL, i.e. gains in longevity are mostly spent in poor health.

Nonetheless, the overall trend in healthy life years is very similar for both measures, indicating that the prevalence is the driving force in HCAL as well as in conventional HE. Albeit we observed gains in total life years for all analysed countries between 2008 and 2014, regardless if measured with period LE or CAL. Many populations still experienced declines in healthy life years in the same period. Naturally, these declines are attributed to increases in the age-specific prevalence of being in the unhealthy state. This finding demonstrates once more the great relevance for accurate health data for analysing healthy life years. Therefore, our results suggest that the health data incorporates large problems as we have shown by unexplainable jumps in the data or outliers. One example for this is the relatively large decrease of about 4 years in conventional HE for Finish females between 2008 and 2014 which is more attributed to random fluctuations in the health data than to real health deteriorations in the Finish population [42]. Last but not the least, the statistical insecurity related to the health data is so high that confidence intervals are not helpful for the analysis. This can be seen from the data presented in Tables 4 and 5. Taking into account these 95% confidence intervals, there are no statistically significant differences between HCAL and conventional HE as well as in the changes in healthy life years over time. This uncertainty results almost entirely from the health data rather than from the mortality data. For this reason, the lack of statistical difference does not imply that the two conceptual approaches are indistinguishable.

We used the GALI health indicator for our empirical applications which refers to a self-reported survey question about longstanding limitations in daily activities. In general, GALI has been validated positively, i.e. GALI is strongly associated with limitations in activities of daily living (such as washing, getting dressed or out of the bed), intermediately associated with limitations in instrumental activity of daily living (such as the need of assistance in doing light housework or managing medication), and somewhat lower association with physical limitations (biting, chewing, or kneeling) in most European countries [43]. Nevertheless, self-reported survey questions might still be influenced by age, culture, and social background of the respondent [44]. For these reasons, the presented trends should be interpreted with caution. In this paper, they served primarily the purpose of demonstrating differences between HCAL and conventional HE with respect to the underlying mortality information. All the discussed conceptual and health data-related issues would apply likewise to other self-reported health indicators from surveys such as EU-SILC.

## Conclusions

HCAL is a summary measure of health and mortality based on the Sullivan method. Using proportions of cohort survivors instead of period mortality rates requires a long time series of mortality data. We have demonstrated several advantages of HCAL which suggest it is an attractive measure for population health. First, HCAL yields a coherent quantity, i.e., combining the health of the real cohort survivors with mortality of the real cohorts. Second, HCAL offers an alternative perspective on health and mortality. Previous approaches have focused either on one single period or on one single cohort. HCAL, on the other hand, is the sum over all cohorts, of the probability surviving and being in good health at the time of observation. In this sense, HCAL is also a measure of population dynamics and, thus, provides new insights into the evolution of the healthy/unhealthy shares in a population.

The empirical analysis suggests that the quality of health data is much more important than the decision between CAL and LE as basis for the total number of life years. We have shown that the prevalence of being in the (un)healthy state varies notably between populations and across time. These differences have by far the strongest impact on healthy life years derived with the Sullivan method. The overall trend in healthy life years is similar in conventional HE and HCAL and taking into account the uncertainty stemming from the health data does not result in statistically significant differences between both indicators.

Nevertheless, conventional HE and HCAL should not be treated as interchangeable as they correspond to two different concepts. The analysis of the gender gap in healthy life years demonstrates that the choice of the survivorship function can indeed affect the result. By taking into account the past mortality experiences of males and females, HCAL gives a more favourable picture for women compared to conventional HE. Also regarding the compression-expression debate we found that the two indicators can suggest different trends as in the case among Italian females. Accordingly, researchers should consider using HCAL especially in applications where period mortality differs strongly from the actual cohort experience. It is important to note, however, that we do not argue that conventional HE should be replaced by HCAL. Yet, given that HCAL is the conceptually more coherent approach, it is worth to be used in addition to conventional HE whenever it is possible to estimate both indicators because it widens the spectrum of empirical analyses and serves for verification of results based on the highly sensitive HE indicator.

### Supplementary information

**Additional file 1.** R code example for estimating HE and HCAL.

## Figures and Tables

**Fig. 5:**
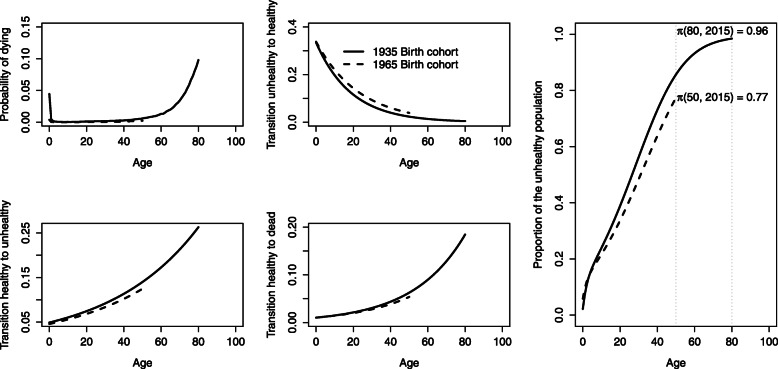
Relationship between transition probabilities and prevalence data. The figure shows simulated age-specific transition probabilities between the states of being healthy, unhealthy, and dead for the cohorts born in 1935 and 1965 (depicted on the left side of the figure). The proportions of the unhealthy population for the birth cohorts 1935 and 1965 are calculated from these age-specific transition probabilities (depicted on the right side of the figure)

## Data Availability

All data used in this paper is publicly available. Mortality data can be found at www.mortality.org and on www.destatis.de for Germany. Health data is available on https://ec.europa.eu/eurostat/web/microdata/european-union-statistics-on-income-and-living-conditions.

## Appendix

**Table 4:** HE and HCAL with 95% confidence intervals (approximated) for five European countries from 2008 to 2014

		2008	2009	2010	2011	2012	2013	2014
**France**								
Females	**HE**	65.11 (± 4.00)	64.91 (± 4.02)	64.82 (± 3.97)	64.74 (± 3.97)	64.76 (± 3.86)	65.01 (± 4.04)	65.29 (± 4.00)
Males		63.22 (± 3.62)	63.02 (± 3.67)	62.97 (± 3.64)	63.16 (± 3.65)	63.13 (± 3.57)	63.22 (± 3.76)	63.50 (± 3.77)
Females	**HCAL**	62.39 (± 3.67)	62.30 (± 3.71)	62.25 (± 3.68)	62.21 (± 3.67)	62.38 (± 3.59)	62.66 (± 3.77)	62.93 (± 3.74)
Males		59.70 (± 3.18)	59.65 (± 3.24)	59.64 (± 3.23)	59.77 (± 3.25)	59.88 (± 3.20)	60.02 (± 3.38)	60.22 (± 3.39)
**Netherlands**								
Females	**HE**	59.14 (± 6.21)	59.17 (± 6.38)	58.77 (± 6.32)	58.08 (± 6.25)	57.70 (± 6.42)	57.30 (± 6.62)	57.31 (± 6.59)
Males		60.43 (± 6.00)	60.60 (± 6.07)	61.25 (± 5.95)	62.18 (± 5.85)	62.48 (± 5.91)	62.59 (± 6.04)	62.41 (± 6.25)
Females	**HCAL**	57.44 (± 5.91)	57.42 (± 6.06)	57.11 (± 6.02)	56.54 (± 5.98)	56.22 (± 6.15)	55.84 (± 6.34)	55.83 (± 6.31)
Males		57.83 (± 5.47)	58.12 (± 5.57)	58.78 (± 5.46)	59.56 (± 5.36)	60.03 (± 5.46)	60.16 (± 5.60)	59.95 (± 5.80)
**Denmark**								
Females	**HE**	59.52 (± 8.31)	59.38 (± 8.32)	59.40 (± 8.37)	59.33 (± 9.23)	59.14 (± 9.05)	58.74 (± 9.07)	58.51 (± 8.92)
Males		61.23 (± 7.56)	61.16 (± 7.62)	60.98 (± 7.61)	60.89 (± 8.19)	60.52 (± 8.31)	59.96 (± 8.44)	59.17 (± 8.28)
Females	**HCAL**	57.08 (± 7.80)	56.97 (± 7.83)	56.91 (± 7.88)	56.72 (± 8.68)	56.57 (± 8.53)	56.24 (± 8.56)	56.01 (± 8.42)
Males		58.13 (± 6.98)	58.07 (± 7.04)	57.94 (± 7.03)	57.71 (± 7.53)	57.34 (± 7.67)	56.86 (± 7.81)	56.23 (± 7.68)
**Germany**								
Females	**HE**	56.32 (± 4.00)	57.01 (± 4.02)	57.30 (± 4.08)	57.12 (± 4.06)	56.40 (± 4.17)	55.35 (± 4.30)	54.43 (± 4.35)
Males		54.50 (± 3.86)	55.40 (± 3.83)	55.89 (± 3.84)	56.10 (± 3.82)	56.12 (± 3.92)	55.71 (± 4.10)	55.00 (± 4.22)
Females	**HCAL**	54.35 (± 3.69)	55.11 (± 3.72)	55.45 (± 3.78)	55.32 (± 3.78)	54.67 (± 3.90)	53.79 (± 4.06)	52.88 (± 4.10)
Males		52.00 (± 3.49)	52.93 (± 3.47)	53.44 (± 3.49)	53.65 (± 3.48)	53.67 (± 3.57)	53.39 (± 3.77)	52.67 (± 3.89)
**United Kingdom**								
Females	**HE**	65.11 (± 4.35)	64.81 (± 4.58)	64.58 (± 4.69)	64.57 (± 4.72)	64.34 (± 4.22)	64.36 (± 4.18)	64.36 (± 4.21)
Males		63.41 (± 4.23)	63.49 (± 4.44)	63.50 (± 4.57)	63.76 (± 4.58)	63.90 (± 4.06)	64.23 (± 3.99)	64.19 (± 4.03)
Females	**HCAL**	62.64 (± 4.07)	62.32 (± 4.28)	62.17 (± 4.40)	62.16 (± 4.44)	62.18 (± 3.97)	62.32 (± 3.94)	62.38 (± 3.97)
Males		60.44 (± 3.88)	60.42 (± 4.07)	60.45 (± 4.20)	60.70 (± 4.21)	61.03 (± 3.73)	61.50 (± 3.68)	61.63 (± 3.72)

The table provides HE and HCAL estimates with 95% confidence intervals for France, the Netherlands, Denmark, Germany, and the UK from 2008 to 2014 (separated for males and females). The confidence intervals are approximated by estimating standard errors for age-specific prevalence values based on the number of individuals in each age interval and are therefore relatively wide for countries and years with small sample size. (Potential uncertainty due to the mortality data is ignored). Source: EU-SILC and HMD data (own calculations)

**Table 5:** HE and HCAL with 95% confidence intervals (approximated) for four European countries in 2008 and 2014

		2008	2014
**Finland**			
Females	**HE**	60.36 (± 6.50)	56.56 (± 6.65)
Males		58.38 (± 5.38)	58.91 (± 5.62)
Females	**HCAL**	58.03 (± 5.96)	54.78 (± 6.26)
Males		55.61 (± 4.81)	56.24 (± 5.12)
**Italy**			
Females	**HE**	62.44 (± 2.75)	63.28 (± 2.95)
Males		63.20 (± 2.37)	63.18 (± 2.60)
Females	**HCAL**	59.39 (± 2.43)	60.79 (± 2.68)
Males		59.19 (± 2.05)	59.79 (± 2.31)
**Norway**			
Females	**HE**	69.26 (± 7.62)	69.19 (± 6.80)
Males		69.91 (± 6.26)	71.18 (± 5.38)
Females	**HCAL**	66.89 (± 7.20)	66.95 (± 6.46)
Males		66.43 (± 5.56)	67.88 (± 4.75)
**Sweden**			
Females	**HE**	64.37 (± 7.38)	72.52 (± 6.67)
Males		64.71 (± 6.05)	73.54 (± 5.22)
Females	**HCAL**	62.39 (± 7.02)	70.44 (± 6.39)
Males		62.00 (± 5.62)	70.55 (± 4.40)

The table provides HE and HCAL estimates with 95% confidence intervals for Finland, Italy, Norway, and Sweden in 2008 and 2014 (separated for males and females). The confidence intervals are approximated by estimating standard errors for age-specific prevalence values based on the number of individuals in each age interval and are therefore relatively wide for countries and years with small sample size. (Potential uncertainty due to the mortality data is ignored). Source: EU-SILC and HMD data (own calculations)

### Cross-sectional prevalence rates from a cohort perspective

The proportions of cohort survivors, *p*_*c*_(*x*, *t* − *x*), can be derived on the basis of cohort- and age-specific survival probabilities. Since death is irreversible, this proportion is monotonically decreasing with age. In other words, the stock of cohort survivors at a given point in time is the product of past survival probabilities. The proportions of the unhealthy population, *π*(*x*, *t*), on the other hand, are more complex in the sense that they are a function of all the past transitions between the states “healthy”, “unhealthy”, and “death” [36]. Unfortunately, detailed data on such transitions are not available for a long time series. Still, cross-sectional health data provides information about the proportion of healthy individuals for a given cohort at a particular point in time as a result of these transitions. For example, a representative health survey conducted in the year 2015 allows us to estimate the fraction of healthy individuals over all individuals for each birth cohort alive in 2015, i.e. cohorts born between 1915 and 2015 (assuming the survey includes the population at age zero to 100). In this way, the exact health trajectories for cohorts remain unobserved but have been implicitly included in the *π*(*x*, *t*) function. This is illustrated in Fig. 5 for the 1935 and 1965 birth cohorts. Guillot and Yu [45] provide an equation for calculating the population proportion of unhealthy individuals at a particular point in time on the basis of transition probabilities between health states and death ([45], p. 508). This equation has been applied to simulated data for the sake of demonstrating the relationship between in- and outflows, i.e. transitions to the (un)healthy state, and the proportion of the unhealthy population as a stock. Please note that assuming an exponential trend for health transition probabilities is in line with previous research [46–48]. The transitions (left panel) produce the prevalence of being unhealthy (right panel). This is 96 percent for the 1935 birth cohort at age 80 (in 2015). The younger 1965 birth cohort turns 50 in 2015 and reaches a proportion of 77% unhealthy individuals. The younger cohort has been exposed to favourable health and mortality conditions (indicated by favourable transition probabilities). This is why a higher share has been found unhealthy at age 50 for the 1935 birth cohort compared to the 1965 birth cohort (over 80% vs. 77%). The observed cross-sectional prevalence at time *t*, i.e. the *π*(*x*, *t*) function, does not provide any information about the exact morbidity and mortality trajectories of cohorts but gives an estimate of the unhealthy population stock for the cohorts reaching age *x* in time *t* (born in time *t − x*) in accordance with their underlying multistate process.

### HCAL and HE in relative terms

Differences in the pattern of the *p*_*c*_(*x*, *t* − *x*)function compared to the *p*(*x*, *t*) affect the outcome of the measure. First, the less rectangular pattern of the *p*_*c*_(*x*, *t* − *x*) function causes lower absolute values for HCAL. Obviously, the lower age-specific survival probabilities result in a lower total number of person-years lived and therefore, HE will exceed HCAL in absolute terms. Interestingly, the choice of the survivorship function also affects the measure in relative terms, i.e. the ratio HCAL/CAL vs. the ratio HE/LE. The reason for that can be revealed by looking at person-years lived in relative terms (dividing the age-specific count of person-years lived by the total number of person-years lived). As already mentioned, the total number of person-years lived is lower in CAL. However, at young ages, both functions show similar number of person-years lived. This is why dividing the healthy life years at young ages by the total number of life years yields greater ratios for HCAL/CAL compared to HE/LE (Fig. 6). This relationship inverses at older ages so that the ratios for HE now exceed the ones for HCAL. In other words, the *p*_*c*_(*x*, *t* − *x*) function (in relative terms) gives more weights on young ages and less weights on old ages compared to the *p*(*x*, *t*) function. Since individuals are mostly healthy at young ages and become increasingly more unhealthy at older ages, the share of healthy life years on total life years is higher in HCAL.

### Interpreting HE and HCAL results

The main purpose of HE is to combine the health and mortality conditions prevailing in one particular period to one single measure. The mortality information in the *p*(*x*, *t*) function refers solely to time *t* so that LE is a pure period mortality measure [10]. What about the *π*(*x*, *t*) function? This function refers to the age-specific prevalence of the population in healthy and unhealthy states at time *t* and, therefore, can be seen as a period estimate as well. In this sense, HE(*t*) is a period measure that reflects the health composition of the real population at time *t* adjusted for period mortality [49]. However, it is not a pure period indicator in a synthetic cohort fashion [5]. We have illustrated that the health state-specific prevalence depends on past mortality rates and health transitions rates. Therefore, HE cannot be interpreted as an average number of healthy life years, lived by currently newborns under the assumption that they are solely exposed to the health and mortality conditions observed in one single period. In order to interpret HE in the same manner as LE, one would need to replace the *observed* prevalence of being in the (un)healthy state with a *constructed* “period” or “equilibrium” prevalence of being in the (un)healthy state [50]. This is a synthetic prevalence calculated from transitions between health states and death observed in a given period. Lièvre et al. [48] have shown that this modelled prevalence is currently lower than the observed prevalence in the USA. Only if health and mortality conditions are constant over time, i.e. constant transition rates, the period prevalence equals the observed prevalence [50]. In this scenario, the Sullivan method yields a period HE estimate, which is equal to the results based on the multistate life table method [51].

CAL differs from period and cohort LE because it does not refer to one single cohort. While period and cohort life tables give an estimate of the average number of person-years lived for one cohort (real or synthetic), CAL is the average number of person-years, which has been lived by all cohorts alive in a given period (assuming a closed population with a constant inflow of annual births). Thus, HCAL refers to the average number of healthy person-years lived by these cohorts. In other words, HCAL reflects the health and mortality conditions that the cohorts present in a population at a given point in time have been exposed to during their past life course. Alternatively, CAL(*t*) can also be seen as the relative population size at time *t* in a constant-birth population [15]. Imagine a population where all cohorts have the same initial size, i.e. a population with a constant number of births each year. Formally, this model population is expressed as follows:

10 $$ N(t)={\int}_0^{\infty }B\cdotp {p}_c\left(x,t-x\right) dx $$

with *N*(*t*) being the total population at time *t* and *B* the number of the annual inflow of births. Rewriting the equation and substituting CAL(*t*) for integrated *p*_*c*_(*x*, *t* − *x*) function yields the population size at time *t* as the product of CAL(*t*) and *B*.

11 $$ N(t)=B\cdotp {\int}_0^{\infty }{p}_c\left(x,t-x\right) dx $$

12 $$ N(t)=B\cdotp CAL(t) $$

Let us assume that the initial size of each cohort is 100,000 (*B* = 100,000). By applying the cohort survivor probabilities (given by the *p*_*c*_(*x*, *t* − *x*) function) to this modelled population, each cohort is exposed to the mortality regime, which actually took place during its past life course (from birth up to time *t*). Since CAL(*t*) summarizes the cohort survivor proportions in time *t*, multiplying the CAL value by 100,000 yields the population size in time *t*. Applying the same population model to HCAL allows to interpret HCAL(*t*) as the relative size of the healthy population in time *t*.

13 $$ {N}^{\mathrm{Healthy}}(t)={\int}_0^{\infty }B\cdotp \mathrm{HCAL} $$

In this perspective, looking at HCAL estimates can be seen as looking at population data, while controlling for fluctuations in births. Comparing CAL and HCAL estimates for a given population over time indicates clearly how the healthy and total share of individuals in a population has evolved. In the case of an increasing HCAL, changes in transition rates promote a situation in which the healthy population is increasing in size over time. This is the clearest sign that people are indeed living longer and healthier lives.

## Abbreviations

CAL: Cross-sectional average length of life
EU-SILC: European Union Statistics on Income and Living Conditions
GALI: Global Activity Limitation Indicator
HCAL: Cross-sectional average length of healthy life
HMD: Human Mortality Database
LE: Life expectancy
WWII: World War II
