# Correction to: The cross-sectional average length of healthy life (HCAL): a measure that summarizes the history of cohort health and mortality

**DOI:** 10.1186/s12963-021-00254-3

**Published:** 2021-04-21

**Authors:** Markus Sauerberg, Michel Guillot, Marc Luy

**Affiliations:** 1 Vienna Institute of Demography, Austrian Academy of Sciences, Vienna, Austria; 2 Wittgenstein Centre for Demography and Global Human Capital (IIASA, OeAW, University of Vienna), Vienna, Austria; 3 Population Studies Center, University of Pennsylvania, Philadelphia, PA USA; 4 French Institute for Demographic Studies (INED), Paris, France

**Correction to: Popul Health Metrics 18, 21 (2020)**

**https://doi.org/10.1186/s12963-020-00220-5**

Following publication of the original article [1], it was several errors were reported in the main text of the article, and also in Table 1.

The heading of Table 1 was missing the symbol ‘π’, and similarly it was missing in the body of the Table for the rows indicating the total prevalence of being unhealthy for the nine European countries. The corrected Table 1 is given below.

**Table 1:** Total sample size N (unweighted) and total prevalence of being unhealthy π (weighted) for nine European countries from 2008 to 2014

			EU-SILC Survey Year						
			2008	2009	2010	2011	2012	2013	2014
**Denmark**	Females	***N***	3019	3101	3072	2655	2737	2784	2959
	Males		2758	2765	2794	2477	2552	2635	2798
	Females	**π**	30.71	30.66	29.59	31.58	30.68	31.70	31.37
	Males		23.65	24.42	23.46	21.93	27.24	26.72	28.57
**Finland**	Females	***N***	5175	5050	5423	4512	4854	5383	5418
	Males		5128	4912	5267	4586	4885	5371	5405
	Females	**π**	34.99	36.17	37.11	37.56	40.82	47.84	39.28
	Males		29.18	29.51	29.18	30.82	32.67	40.61	30.97
**France**	Females	***N***	10,473	10,568	10,944	11,132	11,771	10,803	11,113
	Males		9535	9545	9944	10,164	10,742	9782	9985
	Females	**π**	25.08	26.61	27.23	27.10	26.86	26.99	27.01
	Males		21.07	21.36	23.05	22.47	22.84	22.67	22.41
**Italy**	Females	***N***	22,635	22,072	NA	20,392	20,325	19,039	20,409
	Males		20,741	20,087	NA	18,564	18,475	17,324	18,435
	Females	**π**	31.19	30.35	NA	31.59	32.49	32.95	31.75
	Males		23.62	22.97	NA	24.07	26.21	26.69	25.93
**Germany**	Females	***N***	12,579	12,323	12,191	12,497	12,181	11,671	11,715
	Males		11,547	11,363	11,211	11,548	11,272	10,709	10,780
	Females	**π**	36.44	35.56	35.50	36.14	38.03	38.80	40.17
	Males		33.68	32.82	32.42	33.52	34.87	34.83	36.78
**Netherlands**	Females	***N***	5667	5274	5494	5679	5479	5384	5464
	Males		4648	4443	4628	4794	4667	4706	4680
	Females	**π**	34.14	33.94	33.50	34.79	35.91	39.69	36.74
	Males		24.68	25.81	26.35	23.60	24.73	27.44	24.95
**Norway**	Females	***N***	2632	2587	2457	2054	2820	2838	3490
	Males		2853	2762	2704	2343	3158	3107	3782
	Females	**π**	21.79	21.39	20.03	26.45	17.52	22.07	21.69
	Males		13.40	14.87	14.87	18.54	11.99	14.16	12.49
**Sweden**	Females	***N***	3834	3891	3713	3512	3482	3165	2933
	Males		3612	3649	3451	3193	3136	3025	2834
	Females	**π**	29.29	26.91	26.66	27.88	27.28	27.22	16.95
	Males		21.44	19.45	20.01	21.27	20.82	20.10	10.55
**UK**	Females	***N***	8725	8081	7827	7728	9688	9716	9466
	Males		7816	7278	6970	6949	8648	8692	8437
	Females	**π**	20.74	21.68	22.23	23.55	23.63	23.16	24.52
	Males		18.22	18.91	19.18	19.42	19.70	19.85	21.43

Source: EU-SILC data (own calculations)

In the Results section the following sentences have been corrected, with the text highlighted in bold added.

- For example, Italian males spend 81.06% of their total life years in good health on the basis of HCAL, whereas the proportion of healthy life years is only 78.44% **on the basis of HE**.
- As mentioned above, differences between HE and HCAL in relative terms result from the relative difference between the *p*(*x, t*) function and the *p*_*c*_(*x, t − x*) function, leading to an (un)favorable age-specific weighting scheme **for women**.

In the Discussion section the following sentence has been corrected, with the corrected text highlighted in bold originally erroneously reversed.

- The ratio of **HE/LE** respective **HCAL/CAL** is particularly relevant in this context because it shows the relative share of healthy life years on total life years.

The original text also contained some minor typographical errors which have been amended. The original article has been updated.
