# Supplementary material for: The cross-sectional average length of healthy life (HCAL): a measure that summarizes the history of cohort health and mortality
Source: Popul Health Metr. 2020 Aug 31;18:21. doi: 10.1186/s12963-020-00220-5 (PMC7457804; doi:10.1186/s12963-020-00220-5)
Supplement: Supplementary file 1 — Additional file 1. R code example for estimating HE and HCAL. [file 12963_2020_220_MOESM1_ESM.pdf]

Guide for estimating HE and HCAL

Markus Sauerberg[*]

June 2019

Abstract

This description will allow you to follow the estimation of (smoothed) prevalence rates and LE, HE, CAL, as well as HCAL. The provided R code requires two files: (1) the period life table (fltper_1x1.txt) and (2) the population size (Population.txt) for French females. Both are accessible on the HMD webpage (www.mortality.org). The age-specific prevalence of being in the healthy state has been replaced by simulated data for this example. Consequently, HE and HCAL will differ from the estimates published in the paper. However, LE and CAL should be the same unless the HMD has changed its estimates. These results are based on HMD data downloaded in June 2019.

Contents

Estimating single age-specific prevalence rates **1**

Smoothing single age-specific prevalence rates **2**

Extrapolating prevalence rates **3**

Combining the prevalence rates with LE and CAL **3**

Estimating single age-specific prevalence rates

Our version of EU-SILC data covers the non-institutional population between age 16 and age 80+. In some countries, there was no survey respondent aged 16. Hence, we excluded this age group from our analysis and started to calculated single age-specific prevalence rates starting from age 17 up to age 79 (the highest observed single-age group). The following code will demonstrate our approach based on simulated health survey data and HMD mortality data.

```
# load these packages
library(scales)
library(MortalitySmooth)
library(dplyr)
```

```
# put here the HMD period life table and population data for France
setwd("/home/markus/HCALpaper/Data/France")
# lets simulate some data
x <- 17:79
m <- length(x)
pop <- read.table("Population.txt", header=TRUE, skip=2)
e <- pop$Female[pop$Year==2014][x+1]
e <- e/1000
off <- log(e)
# log-prevalance
etaT <- seq(log(0.1), log(0.7), length=m)
etaT <- rescale(etaT, c(-4, -0.5))
muT <- exp(etaT)
```

*Vienna Institute of Demography, Vienna, Austria, markus.sauerberg@oaew.ac.at.

```
# simulate counts
yT <- e*muT
y <- rpois(m, yT)
lmx <- log(y/e)
```

Smoothing single age-specific prevalence rates

Please see Camarda (2012)[1] for more details.

```
# smoothing
fit0 <- Mort1Dsmooth(x=x, y=y,
                     offset=off,
                     method=3, lambda=10^1)

etaS <- fit0$logmortality

# plot
true.prev <- 1-muT
observed.prev <- 1-exp(lmx)
smoothed.prev <- 1-exp(etaS)

plot(x, observed.prev, ylim=c(0,1), xlab="Age", ylab="Proportion healthy")
lines(x, true.prev, col=2)
lines(x, smoothed.prev, col=3)
legend("bottomleft", legend=c("observed","true","smoothed"),
       lwd=1, col=c(1,2,3), bty="n"
       )
```

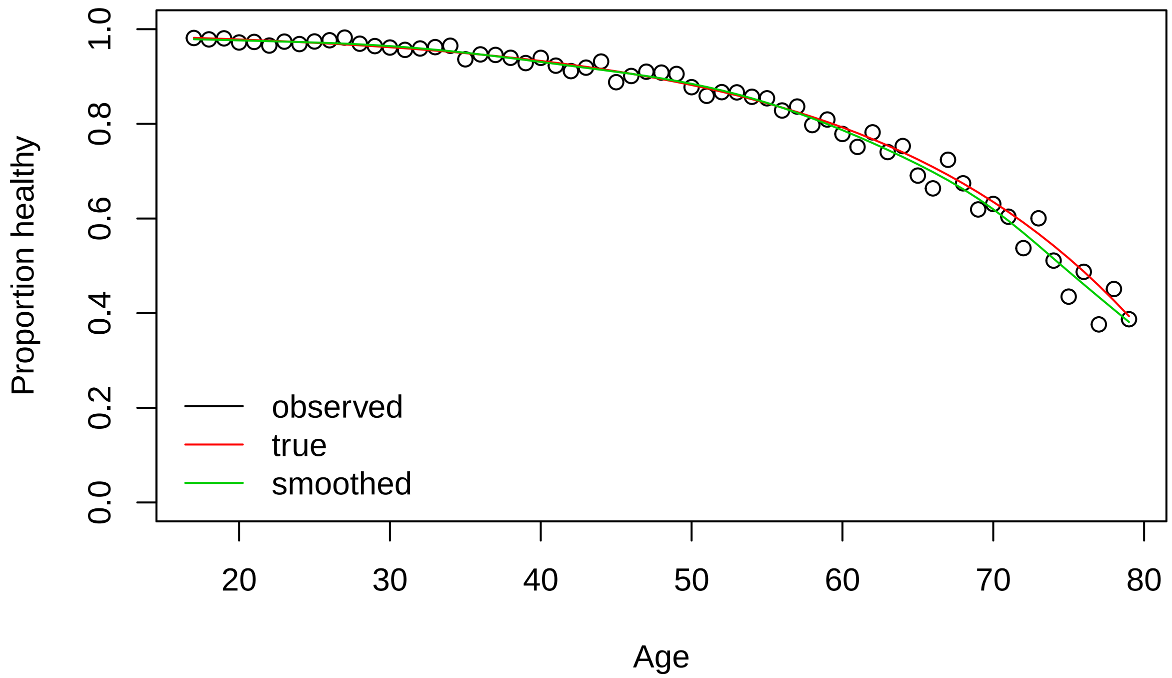

[1] Camarda, C.G. (2012) MortalitySmooth: An R Package for Smoothing Poisson Counts with P-Slines, Journal of Statistical Software.

Extrapolating prevalence rates

We are only able to observe the health state-specific prevalence between ages 16 and 79. In order to estimate HCAL and HE we extrapolate the data based on a modified version of the EUROSTAT[2] and EUROHEX[3] method. They group the data for the $16-19$ age interval and assume that the prevalence for all individuals before age 16, i.e., $(0-15)$, is half of it. However, we defined the prevalence of a new-born as half of the prevalence for an individual aged 17 and assumed an exponential trend between these two ages. Similarly, we extrapolated age-specific prevalence rates for people over 79 years by defining the observed prevalence at age 85+ (downloaded from eurohex.eu) as the prevalence at age 100 and assumed again an exponential trend between these two ages. Note, that there is a trad-eoff between closing the life table at an earlier age, e.g., at age 85, and making assumptions about the unobserved health state-specific prevalence in very old ages. We decided to extrapolate up to age 100 because our main aim is to assess the affect of the underlying mortality function, i.e., $p(x,t)$ and $p_c(x,t-x)$. Truncating these functions already at age 85 would result in a great loss of mortality information. Additionally, the person-years lived in very old ages are relatively small so that the choice of the proportions of the population in poor health contribute comparatively less to the overall count of healthy person-years lived.

```
open.age.prev <- c(0.26) #Prevalence at age 85+, obtained from www.eurohex.eu

Prev <- c(
    exp(
    seq(log(1-((1-smoothed.prev[1])/2)),
    log(smoothed.prev[1]),
    length.out=18)[1:17]),
    smoothed.prev[1:62],
    exp(seq(log(smoothed.prev[63]), log(open.age.prev), length=22))
)
```

Combining the prevalence rates with LE and CAL

In the next step, we calculate CAL, HCAL, LE, and HE for French females in 2014. This following code gives all estimates based on the person-years lived approach (Equations 4,5,6 and 7) as well as solely based on the (weighted) survivorship functions (Equation 2,3,8, and 9).

```
# put here the HMD period life table and population data for France
setwd("/home/markus/HCALpaper/Data/France")
females <- read.table("fltper_1x1.txt",header=TRUE,fill=TRUE,skip=1)
period <- 2014
females$Age <- as.numeric(as.character(females$Age))
females$Age[is.na(females$Age)] <- 110
females$px <- 1-females$qx
femalesLT <- females
females <- females[,c("Year", "Age", "px", "Lx")]
females$Cohort <- females$Year-females$Age
females <- arrange(females, Cohort)
females <- females %>% group_by(Cohort) %>%
              mutate(pxC=cumprod(px)) %>%
              mutate(lx=c(1, cumprod(px)[1:length(px)-1])) %>%
              mutate(dx=c(-diff(lx), lx[length(lx)])) %>%
              mutate(ax=ifelse(Age==0, 0.14, 0.5)) %>%
              mutate(Lx=c(lx[-1]+ax[-length(ax)]*dx[-length(dx)],NA))
```

[2] https://ec.europa.eu/eurostat/cache/metadata/Annexes/hlth__hlye__esms__an1.pdf
[3] http://www.eurohex.eu/

```r
females.select <- filter(females, Cohort>=(period-100) & Cohort<=period & Year<=period)

CALpx <- c()

for (i in 1:101) {
     CALpx[i] <- females$pxC[females$Cohort==(period+1-i) & females$Age==(i-1)]
      }

CALLx <- c()

for (i in 1:101) {
     CALLx[i] <- females$Lx[females$Cohort==(period+1-i) & females$Age==(i-1)]
      }

###CAL and HCAL###
CAL.bypx <- sum(CALpx)+0.5
CAL.byLx <- sum(CALLx)
print(cbind(CAL.bypx,CAL.byLx),4)
```

```
##      CAL.bypx CAL.byLx
## [1,]    80.75    80.59
```

```r
HCAL.bypx <- sum(CALpx*Prev)+0.5
HCAL.byLx <- sum(CALLx*Prev)
print(cbind(HCAL.bypx,HCAL.byLx),4)
```

```
##      HCAL.bypx HCAL.byLx
## [1,]     67.43     67.07
```

```r
###LE and conventional HE###
px.period <- 1-femalesLT$qx[femalesLT$Year==period]
Lx.period <- femalesLT$Lx[femalesLT$Year==period][c(1:101)]
px.cumu <- cumprod(px.period[c(1:101)])
LE.bypx <- sum(px.cumu)+0.5
LE.byLx <- sum(Lx.period/100000)
print(cbind(LE.bypx, LE.byLx), 4)
```

```
##      LE.bypx LE.byLx
## [1,]   85.37   85.36
```

```r
HE.bypx <- sum(px.cumu*Prev)+0.5
HE.byLx <- sum(Lx.period/100000*Prev)+0.5
print(cbind(HE.bypx, HE.byLx), 4)
```

```
##      HE.bypx HE.byLx
## [1,]   69.82      70
```
